# Lack of H3K27 trimethylation is associated with 1p/19q codeletion in diffuse gliomas

**DOI:** 10.1007/s00401-019-02025-9

**Published:** 2019-05-07

**Authors:** Katharina Filipski, Yannick Braun, Jenny Zinke, Bastian Roller, Peter Baumgarten, Marlies Wagner, Christian Senft, Pia S. Zeiner, Michael W. Ronellenfitsch, Joachim P. Steinbach, Karl H. Plate, Gilles Gasparoni, Michel Mittelbronn, David Capper, Patrick N. Harter

**Affiliations:** 1 Institute of Neurology, (Edinger Institute), University Hospital, Frankfurt Am Main, Germany; 2 German Cancer Consortium (DKTK), Partner Site Frankfurt/Mainz, Heidelberg, Germany; 3 German Cancer Research Center (DKFZ), Heidelberg, Germany; 4 Department of Neurosurgery, University Hospital, Frankfurt Am Main, Germany; 5 Institute of Neuroradiology, University Hospital, Frankfurt Am Main, Germany; 6 Department of Neurology, University Hospital, Frankfurt Am Main, Germany; 7 Dr. Senckenberg Institute of Neurooncology, University Hospital, Frankfurt Am Main, Germany; 8 Frankfurt Cancer Institute (FCI), Frankfurt Am Main, Germany; 9 Department of Genetics, University of Saarland, Campus A2 4, Saarbrücken, Germany; 10 Luxembourg Centre of Neuropathology (LCNP), Luxembourg City, Luxembourg; 11 Luxembourg Centre for Systems Biomedicine (LCSB), University of Luxembourg, Luxembourg City, Luxembourg; 12 Laboratoire National de Santé (LNS), Dudelange, Luxembourg; 13 NORLUX Neuro-Oncology Laboratory, Luxembourg Institute of Health (LIH), Luxembourg, Luxembourg; 14 Charité-Universitätsmedizin Berlin, Corporate Member of Freie Universität Berlin, Humboldt-Universität zu Berlin, Berlin, Germany; 15 Department of Neuropathology, Berlin Institute of Health, Berlin, Germany; 16 German Cancer Consortium (DKTK), Partner Site Berlin, German Cancer Research Center (DKFZ), Heidelberg, Germany

The current World Health Organisation (WHO) Classification of Central Nervous System Tumours defines oligodendrogliomas by IDH mutation and 1p/19q codeletion [12]. Oligodendrogliomas differ from diffuse astrocytomas regarding genetic alterations in telomere maintenance mechanisms by frequently displaying TERT promoter mutations while astrocytomas typically exhibit ATRX (a-thalassaemia/mental retardation syndrome X-linked) mutations leading to alternative lengthening of telomeres (ALT) [1, 2, 7, 10, 13]. The distinction between both glioma types is crucial since it has a considerable impact on both patient treatment and outcome.

Currently, methodological guidelines for the assessment of 1p/19q codeletion are missing and commonly PCR-based loss of heterozygosity analyses or fluorescent in situ hybridization (FISH) are used. IDH mutant astrocytomas and oligodendrogliomas can also be distinguished by DNA methylation-based profiling, which allows 1p/19q assessment via calculated copy number profiles (CNP) [4]. Infinium 850 k EPIC array is a highly reliable and accurate technique for DNA methylation analysis but can currently only be performed by specialized laboratories as it requires substantial investment in infrastructure and personnel.

Trimethylation at lysine 27 of histone 3 (H3K27me3) is a repressive histone mark associated with inhibition of transcription and represents a post-translational modification set by EZH2, a component of the Polycomb repressive complex 2 [5, 15, 16]. In a paediatric ependymoma cohort, reduction of H3K27me3 defines a subgroup of posterior fossa ependymomas with poor prognosis [3, 17]. Likewise, WHO grade I and II meningiomas display a higher risk of recurrence when H3K27me3 is lost [9]. However, since comprehensive data about H3K27me3 in IDH mutant gliomas is missing, we screened an epigenetically well-defined glioma cohort consisting of 26 IDH mutant and 1p/19q codeleted oligodendrogliomas, 34 IDH mutant astrocytomas and 101 IDH wildtype glioblastomas for potential differences in H3K27 trimethylation on protein level.

Diffuse gliomas showed differences in H3K27me3 staining by either displaying nuclear retention or lack of H3K27me3 immunoreactivity (Fig. 1a). In line with H3K27me3 functioning as a transcriptional silencing mechanism via chromatin remodelling for X-inactivation, we also noticed nuclear lack of H3K27me3 in combination with preserved dot-like staining of the inactivated X chromosome in a subgroup of female glioma patients (Fig. 1a). Furthermore, H3K27me3 turned out to be an indicator of patient survival: patients with lack of H3K27me3 expression showed a significantly better prognosis than patients with retained H3K27me3 staining (Supplementary Fig. 1).

**Fig. 1:**
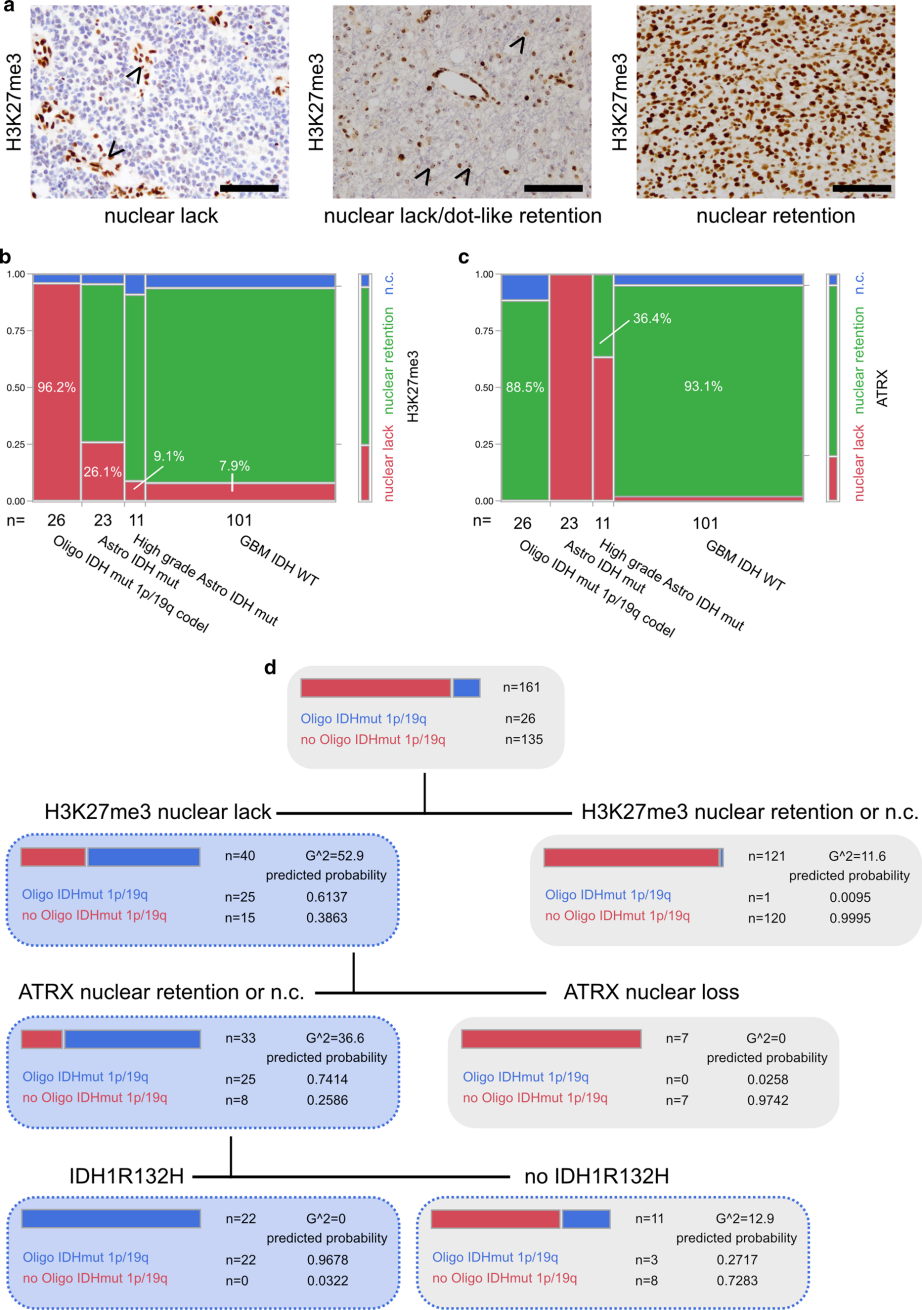
**a** Immunohistochemistry against H3K27me3. Arrowheads pointing at retained nuclear staining in endothelial cells while tumour cells show a lack of staining (IDH mutant and 1p/19q codeleted oligodendroglioma, left panel). Arrowheads pointing at dot-like H3K27me3 retention in otherwise H3K27me3-negative tumour cells in a case of a female patient (IDH mutant and 1p/19q codeleted oligodendroglioma, middle panel). Nuclear retention of H3K27me3 in a glioblastoma IDH-WT specimen (right panel) (all scale bars 100 µm). **b** H3K27me3 and **c** ATRX expression in epigenetically defined glioma subclasses [4] (*n.c.* non-conclusive). **d** Decision tree of recursive partitioning using DNA methylation-based classifier diagnosis “IDH mutant and 1p/19q codeleted oligodendroglioma” as dependent variable and H3K27me3, ATRX and IDH1R132H staining as predictors. Decision tree showing results of best split

Next, we set out to compare H3K27me3 immunoreactivity with DNA methylation classes. None of the investigated tumours showed a histone 3 lysine 27 M mutation, as assessed by a mutation-specific antibody (Supplementary Fig. 1). Most interestingly, in IDH mutant and 1p/19q codeleted oligodendroglioma, H3K27me3 retention was never observed, the abundant number of cases (25/26) showed clear lack of nuclear H3K27me3 staining and only one case had to be deemed non-conclusive (Fig. 1b). Gliomas belonging to the DNA methylation class of IDH mutant astrocytomas or IDH mutant high-grade astrocytomas were characterized by nuclear H3K27me3 retention in 69.6% and 81.8%, respectively (Fig. 1b). Malignant gliomas of the DNA methylation class of IDH wildtype glioblastoma predominantly presented with preserved nuclear H3K27me3 staining (86.1%, Fig. 1b). Interestingly, in IDH wildtype glioblastomas, lack of nuclear staining was exclusively associated with methylation subclasses “mesenchymal” (3 out of 36) and “RTK I” (5 out of 26) (Supplementary Fig. 1c displaying examples of H3K27me3 staining in different glioma subtypes). As loss of nuclear ATRX staining and 1p/19q codeletion are mostly mutually exclusive, ATRX immunohistochemistry is useful in discrimination between an oligodendroglial and astrocytic tumour lineage [6, 8, 11, 18]. Nevertheless, non-conclusive ATRX staining impeded diagnosis of oligodendroglioma in 11.5% and might have been misleading in up to 36.4% of IDH mutant high-grade astrocytomas which presented with retained nuclear ATRX staining (Fig. 1c).

To assess the predictive value of H3K27me3 expression in diffuse gliomas we deployed a recursive partitioning model for the prediction of the DNA methylation class IDH mutant and 1p/19q codeleted oligodendroglioma with H3K27me3, ATRX and IDH1R132H staining as predictors (Fig. 1d). Sequential immunohistochemistry against the aforementioned antigens revealed that diffuse gliomas with lack of nuclear H3K27me3 staining, retention or non-conclusive nuclear ATRX staining and IDH1R132H mutation are 1p/19q codeleted oligodendrogliomas with a predicted probability of 0.9678 (Fig. 1d, see also results of a validation cohort in Supplementary Fig. 2).

Our results point to differences in histone H3K27 modification in diffuse glial tumours which are advantageous for the clinically relevant discrimination between astrocytic and oligodendroglial tumour lineage. Results of our prediction model suggests the usage of an antibody panel including antibodies against IDH1R132H, H3K27M (exclusion of mutation), ATRX and H3K27me3 for the prediction of the DNA methylation class “oligodendroglioma, IDH mutant and 1p/19q codeleted” (Supplementary Table 1). In diffuse gliomas harbouring a lack of nuclear H3K27me3 in addition to retained or non-conclusive ATRX staining but no IDH1R132H-mutation, IDH sequencing should be followed up. Small tumour biopsies or infiltration zones require careful evaluation of immunostainings (Supplementary Fig. 1c).

With regard to tumour biology, the lack of nuclear H3K27me3 in oligodendrogliomas is surprising given the fact that increasing levels of 2-hydroxyglutarate in cells harbouring an IDH mutation are known to impair demethylation of other repressive histone marks, such as H3K9me3, with resulting gain of histone methylation [14]. The resulting chromatin compaction would promote CpG island hypermethylation phenotype [19]. The relation between 1p/19q codeletion and global lack of H3K27me3 requires further investigation.

## Electronic supplementary material

Below is the link to the electronic supplementary material.

**Supplementary Figure 1:** (a) Kaplan-Meier survival analyses of a non-molecularly classified tissue micro array (TMA) cohort (1998-2011) of different diffuse gliomas including oligodendrogliomas, astrocytomas, and glioblastomas. (b) Results of IDH1R132H, ATRX, H3K27me3 and H3K27M immunohistochemistry in epigenetically defined glioma subclasses. (c) Collection of H3K27me3 staining patterns in different glioma subtypes (TIFF 8558 kb)

**Supplementary Figure 2:** Immunohistochemical analyses for IDH1R132H, ATRX, H3K27me3 and H3K27M in the epigenetically classified validation cohort consisting of 18 IDH-mutant gliomas (1p/19q codeleted oligodendrogliomas (n=9); astrocytomas (n=9)) (TIFF 1556 kb)

**Supplementary Table 1:** Antibodies, supplier and dilution for immunohistochemistry (DOCX 12 kb)
