# Supplementary figures and images for: Lack of H3K27 trimethylation is associated with 1p/19q codeletion in diffuse gliomas
Source: Acta Neuropathol. 2019 May 7;138(2):331–4. doi: 10.1007/s00401-019-02025-9 (PMC6660498; doi:10.1007/s00401-019-02025-9)

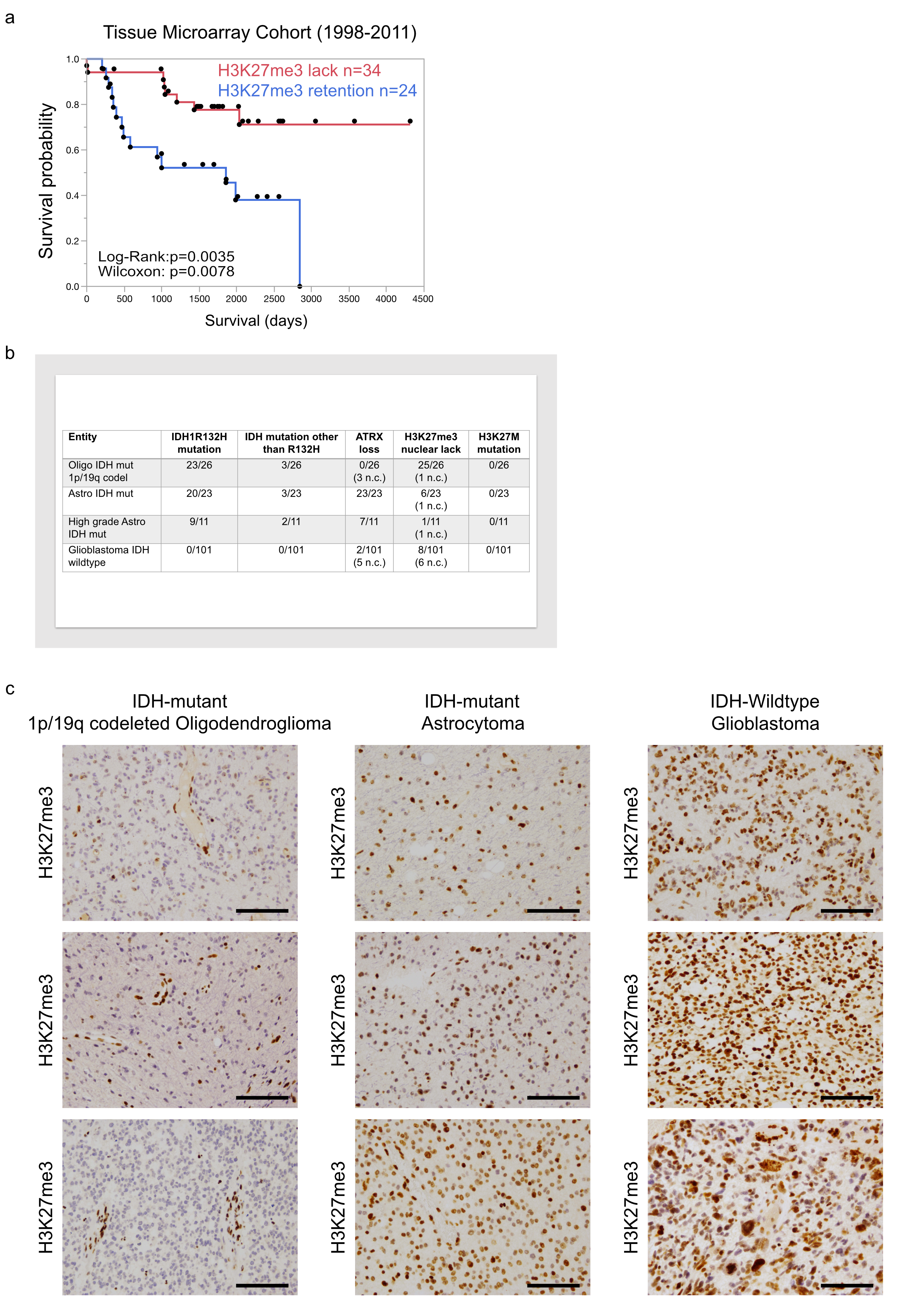

Supplement: Supplementary file 1 — Supplementary Figure 1: (a) Kaplan-Meier survival analyses of a non-molecularly classified tissue micro array (TMA) cohort (1998-2011) of different diffuse gliomas including oligodendrogliomas, astrocytomas, and glioblastomas. (b) Results of IDH1R132H, ATRX, H3K27me3 and H3K27M immunohistochemistry in epigenetically defined glioma subclasses. (c) Collection of H3K27me3 staining patterns in different glioma subtypes (TIFF 8558 kb) [file 401_2019_2025_MOESM1_ESM.tiff]

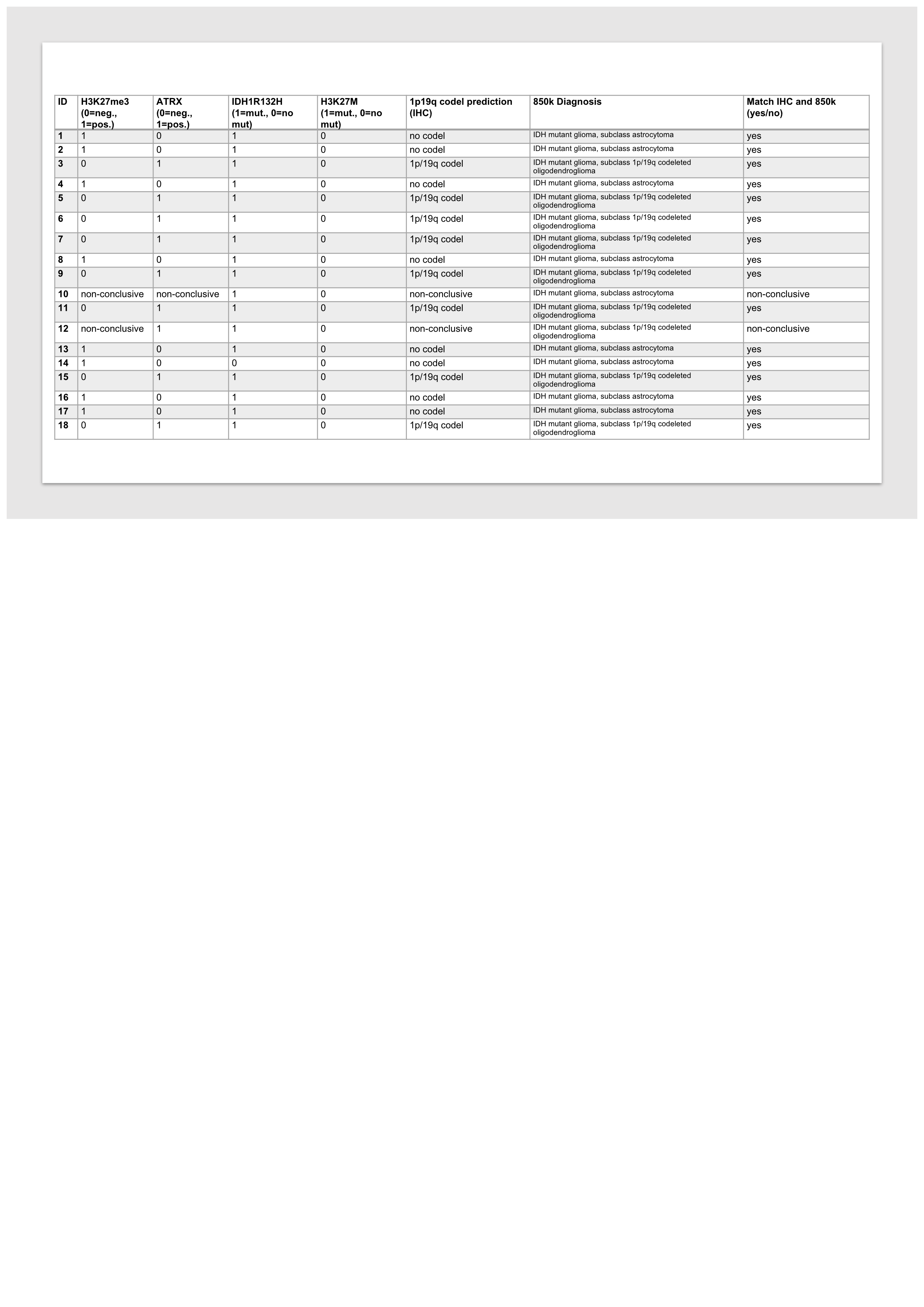

Supplement: Supplementary file 2 — Supplementary Figure 2: Immunohistochemical analyses for IDH1R132H, ATRX, H3K27me3 and H3K27M in the epigenetically classified validation cohort consisting of 18 IDH-mutant gliomas (1p/19q codeleted oligodendrogliomas (n=9); astrocytomas (n=9)) (TIFF 1556 kb) [file 401_2019_2025_MOESM2_ESM.tiff]
