# Supplementary material for: Lack of H3K27 trimethylation is associated with 1p/19q codeletion in diffuse gliomas
Source: Acta Neuropathol. 2019 May 7;138(2):331–4. doi: 10.1007/s00401-019-02025-9 (PMC6660498; doi:10.1007/s00401-019-02025-9)
Supplement: Supplementary file 3 — Supplementary Table 1: Antibodies, supplier and dilution for immunohistochemistry (DOCX 12 kb) [file 401_2019_2025_MOESM3_ESM.docx]

| **Antigen** | **Company** | **Clone** | **Dilution** |
| --- | --- | --- | --- |
| H3K27M | RevMAb Biosciences | RM192 | 1:10000 |
| H3K27me3 | Cell Signaling Technology | C36B11 | 1:200 |
| ATRX | BioSB | BSB-108 | 1:200 |
| IDH1R132H | Dianova | H09 | 1:50 |
